# Extracting immunological and clinical heterogeneity across autoimmune rheumatic diseases by cohort-wide immunophenotyping

**DOI:** 10.1136/ard-2023-224537

**Published:** 2023-10-30

**Authors:** Hiroaki Tanaka, Yukinori Okada, Shingo Nakayamada, Yusuke Miyazaki, Kyuto Sonehara, Shinichi Namba, Suguru Honda, Yuya Shirai, Kenichi Yamamoto, Satoshi Kubo, Katsunori Ikari, Masayoshi Harigai, Koshiro Sonomoto, Yoshiya Tanaka

**Affiliations:** 1 First Department of Internal Medicine, University of Occupational and Environmental Health, Kitakyushu, Fukuoka, Japan; 2 Department of Statistical Genetics, Osaka University School of Medicine Graduate School of Medicine, Suita, Osaka, Japan; 3 Department of Genome Informatics, Graduate School of Medicine, the University of Tokyo, Tokyo, Japan; 4 Laboratory for Systems Genetics, RIKEN Center for Integrative Medical Sciences, Yokohama, Japan; 5 Laboratory of Statistical Immunology, Immunology Frontier Research Center (WPI-IFReC), Osaka University, Suita, Japan; 6 Premium Research Institute for Human Metaverse Medicine (WPI-PRIMe), Osaka University, Suita, Japan; 7 Department of Rheumatology, Department of Internal Medicine, Tokyo Women's Medical University, Shinjuku-ku, Tokyo, Japan; 8 Department of Respiratory Medicine and Clinical Immunology, Osaka University Graduate School of Medicine, Suita, Japan; 9 Laboratory of Children’s health and Genetics, Division of Health Science, Osaka University Graduate School of Medicine, Suita, Japan; 10 Department of Pediatrics, Osaka University Graduate School of Medicine, Suita, Japan; 11 Department of Orthopedics, Tokyo Women's Medical University, Tokyo, Japan; 12 Department of Clinical Nursing, School of Health Sciences, University of Occupational and Environmental Health, Kitakyushu, Japan

**Keywords:** Rheumatoid Arthritis, Lupus Erythematosus, Systemic, Sjogren's Syndrome, Spondylitis, Ankylosing, Scleroderma, Systemic

## Abstract

**Objective:**

Extracting immunological and clinical heterogeneity across autoimmune rheumatic diseases (AIRDs) is essential towards personalised medicine.

**Methods:**

We conducted large-scale and cohort-wide immunophenotyping of 46 peripheral immune cells using Human Immunology Protocol of comprehensive 8-colour flow cytometric analysis. Dataset consisted of >1000 Japanese patients of 11 AIRDs with deep clinical information registered at the FLOW study, including rheumatoid arthritis (RA) and systemic lupus erythematosus (SLE). In-depth clinical and immunological characterisation was conducted for the identified RA patient clusters, including associations of inborn human genetics represented by Polygenic Risk Score (PRS).

**Results:**

Multimodal clustering of immunophenotypes deciphered underlying disease-cell type network in immune cell, disease and patient cluster resolutions. This provided immune cell type specificity shared or distinct across AIRDs, such as close immunological network between mixed connective tissue disease and SLE. Individual patient-level clustering dissected patients with AIRD into several clusters with different immunological features. Of these, RA-like or SLE-like clusters were exclusively dominant, showing immunological differentiation between RA and SLE across AIRDs. In-depth clinical analysis of RA revealed that such patient clusters differentially defined clinical heterogeneity in disease activity and treatment responses, such as treatment resistance in patients with RA with SLE-like immunophenotypes. PRS based on RA case–control and within-case stratified genome-wide association studies were associated with clinical and immunological characteristics. This pointed immune cell type implicated in disease biology such as dendritic cells for RA-interstitial lung disease.

**Conclusion:**

Cohort-wide and cross-disease immunophenotyping elucidate clinically heterogeneous patient subtypes existing within single disease in immune cell type-specific manner.

**WHAT IS ALREADY KNOWN ON THIS TOPIC**

- There exist clinical and immunological heterogeneity across the autoimmune rheumatic diseases (AIRDs).
- Immunophenotypes can dynamically describe immune profiles of individuals with least invasion and thus considered as a promising tool of personalised medicine.
- Inborn genetics highlighted by Polygenic Risk Score (PRS) can predict the risk of AIRDs.

**WHAT THIS STUDY ADDS**

- Our immunophenotyping identified cellular networks linked to particular AIRDs providing immune cell-type specificity that was shared or distinct among the AIRDs.
- Patient-level clustering elucidated immunological polarisation between rheumatoid arthritis and systemic lupus erythematosus across AIRDs, which reflected clinical heterogeneity in disease activity and treatment responses of AIRDs.
- PRS projection into immunophenotypes suggested candidate key immune cell types implicated in disease aetiology.

**HOW THIS STUDY MIGHT AFFECT RESEARCH, PRACTICE OR POLICY**

- Cohort-wide and cross-disease immunophenotyping can elucidate clinical and immunological heterogeneity across the patients with AIRD, which could be a useful approach to implement personalised medicine.

## Introduction

Autoimmune rheumatic diseases (AIRDs) are systemic diseases characterised by orchestration of disrupted self-tolerance of immune systems, including rheumatoid arthritis (RA) and systemic lupus erythematosus (SLE).^1 2^ Given shared immunological backgrounds across AIRDs, glucocorticoid and immunosuppressive drugs have been conventionally used for treatment. Recently, new treatments such as molecular-targeted therapy with biological or targeted synthetic disease-modifying antirheumatic drugs (b/tsDMARDs) or JAK inhibitors are successfully indicated for multiple AIRDs.^3^ However, we face a challenge in characterising clinical immune features and selecting best treatment among multiple targeted therapies for a particular AIRD case towards personalised medicine.^4–6^ Namely, there exist fundamental but not-yet-solved questions of AIRD aetiology; how should we categorise clinical subtypes of AIRDs, define their heterogeneity within and across AIRDs, and plan effective treatment strategy targeting clinical features of AIRD subtypes? Human genetics of AIRDs provided insights on prominent roles of Polygenic Risk Score (PRS) based on the genome-wide association studies (GWAS).^7 8^ This achieved unsupervised clustering of AIRDs from genetic backgrounds^9^ and enabled prediction of a lifetime AIRD risk of individuals.^8^ However, genetics-based prediction is currently neither modelling underlying clinical heterogeneity within AIRDs, nor reflecting progressing immune profiles in a case.

Immunophenotypes, immune-related cell types in peripheral blood mononuclear cells (PBMCs) quantified by flow-cytometry analysis, can dynamically and quantitatively describe immune profiles of individuals. Flow-cytometry is useful to distinguish: (1) differentiation stage such as naïve T cells or memory T cells, (2) lineage or functional difference such as Th1, Th2 or Th17, (3) activation status or cellular signalling pathways of lymphocytes, which often reflect pathological processes in organs/tissue. Further, immunophenotypes are assayed in a less invasive manner without conflicting the ethical issues of individual identification like genome sequencing. They have advantages in deciphering associations with clinical features of AIRDs such as disease activity and responsiveness to bDMARDs,^10–13^ being considered as a promising tool towards personalised medicine of AIRD. Despite its utility, however, existing research of immunophenotypes are limited in their sample sizes, coverage of AIRD spectrum and cell types. Further, lack of the standardisation protocol in immunophenotyping assays have made pan-disease or cross-cohort analysis to be difficult.

Here, we introduce our large-scale and cohort-based immune phenotypes dataset that includes over 1000 individuals of a variety of AIRDs and healthy donors attached to deep and longitudinal clinical information. Our aim is to elucidate complex immunonetwork of AIRDs via the multimodal resolutions of immune cells, diseases and individual patients. As principal strategy, immunophenotypes and human genetics are two major components representing the hypothesis-free approaches to elucidate human immunology (online supplemental figure 1). By using a subset of the dataset including 11 AIRDs and healthy controls with immunophenotypes of the 46 cell types, we revealed immune cell type networks associated with AIRDs in a disease-specific manner, as well as shared and distinct immunological features across AIRDs. Unsupervised learning of immunophenotypes successfully dissected the AIRD cases into several clusters, where heterogeneity within a single AIRD, as well as shared architecture across multiple AIRDs, are revealed. By adopting RA as a flagship disease, we found that patient subtypes defined by immunophenotypes could be characterised by different clinical features. Further, immunophenotypes and clinical predisposition of the patients with RA were linked to inborn human genetics.

10.1136/ard-2023-224537.supp1 Supplementary data

## Methods

### Study participants

We used the data collected through the FLOW study managed by the First Department of Internal Medicine, University of Occupational and Environmental Health, Japan (UOEH). The FLOW study is a prospective cohort recruiting the patients diagnosed with a variety of AIRDs.^10–13^ In this study, 1088 cases affected with any of the 11 AIRDs registered from May 2011 to December 2018, including RA, SLE, systemic sclerosis (SSc), ANCA-related vasculitis (AAV), idiopathic inflammatory myopathy (IIM), psoriasis, IgG_4_-related disease (IgG_4_RD), mixed connective tissue disease (MCTD), ankylosing spondylitis (AS), Sjogren’s syndrome (SjS), and giant cell aortitis (GCA), and 64 controls in the UOEH hospital and the affiliated hospitals were enrolled (table 1). All the patients were diagnosed by professional physicians according to the clinical criteria.^14–25^ A part of the patients with RA was also registered at the FIRST registry, a prospective cohort longitudinally observing the patients with RA who were newly prescribed with b/tsDMARDs.^26 27^ The patients affected with the multiple target AIRDs were excluded, while secondary SjS were included considering its relatively high complication rates with the primary AIRDs.

**Table 1:** Characteristics of the subjects with immunophenotypes

Disease (abbreviation)	No subjects		Sex (female)	Age (median, IQR)	Disease duration (median, IQR)	Disease-specific descriptions (after QC)
	Before QC	After QC				
Rheumatoid arthritis	336	285	84.2%	63.92 (53.84–72.46)	3.75 (1.41–9.80)	ACPA+225/285, RF+238/283, SS-A+54/264, SS-B+10/86
Systemic lupus erythematosus	218	170	88.2%	38.84 (29.53–53.01)	6.04 (1.28–14.20)	Sm+53/164, dsDNA+96/167, SS-A+91/161, SS-B+27/134
Systemic sclerosis	146	131	90.1%	67.26 (55.52–76.41)	2.85 (1.13–8.34)	Centromere+81/121, Scl-70+20/121, RNA polymerase III+11/112
ANCA-related vasculitis	119	94	57.4%	72.04 (66.00–78.46)	1.03 (0.56–2.61)	MPO-ANCA+73/89, PR3-ANCA+15/85
Idiopathic inflammatory myopathy	96	77	70.1%	59.35 (46.10–68.46)	1.19 (0.68–3.15)	ARS+29/61, Jo-1+10/39, MDA5+9/22, TIF1-gamma+2/18
Psoriasis	47	35	28.6%	55.99 (44.18–65.02)	11.62 (6.44–20.78)	
IgG_4_ related disease	41	35	45.7%	67.20 (56.66–73.09)	1.49 (0.76–3.17)	IgG_4_ above reference level 33/35
Mixed connective tissue disease	30	22	86.4%	49.00 (40.00–57.19)	2.69 (0.96–5.66)	RNP+22/22, Sm+5/21
Ankylosing spondylitis	21	13	38.5%	50.75 (47.29–62.01)	9.29 (3.62–20.73)	
Sjogren’s syndrome	21	20	100.0%	58.97 (39.44–66.33)	3.13 (1.54–4.81)	SS-A+17/20, SS-B+11/19
Giant cell aortitis	13	11	81.8%	72.18 (68.26–78.72)	1.59 (0.80–3.80)	
Controls	64	54	70.4%	48.96 (36.63–64.95)		
Total	1152	947	77.4%	60.74 (46.00–71.63)	2.89 (1.09–9.66)	

ACPA, anticitrullinated protein antibody; QC, quality control; RF, rheumatoid factor.

### Immunophenotyping

Immunophenotypes of the 46 immune cell types of the participants were longitudinally generated by analysing flow cytometry of PBMCs according to the protocol described elsewhere.^10 11^ Briefly, after separation and antibody staining (online supplemental table 1), PBMCs were analysed by multicolor flow cytometry (FACSVerse; BD Bioscience). The phenotypes of immune cell subsets were defined based on the Human Immunology Protocol of comprehensive 8-colour flow cytometric analysis proposed by National Institutes of Health/Federation of Clinical Immunology Sciences,^28^ with minor modifications for detecting Tfh cells. Details of the gating strategy are in online supplemental figure 2. After quality control (QC), immunophenotypes of the 947 patients were obtained.

To normalise potential batch effects among the samples and assays, we developed a pipeline to apply automated counting of the immune cells using *R* Bioconductor packages of openCyto (version 2.2.0). The list of the 46 cell types is shown in online supplemental table 2. Counts of each cell and sample were log transformed after normalised by the total viable PBMC counts (ie, sum of the CD4^+^ T cells, CD3^-^CD19^+^ B cells, CD3^−^CD14^−^CD19^−^CD20^−^CD56^+^ NK cells, CD3^−^CD14^−^CD19^−^CD20^−^CD56^−^HLA-DR^+^ dendric cells (DCs) and CD14^+^ monocytes). Details of the immunophenotyping including sample collection and immune cell sorting are described in online supplementary methods.

### Statistical analysis of the immunophenotypes

Statistical analysis of the immunophenotypes was conducted by using *R* statistical software (V.4.0.2), as described elsewhere.^29^ This included Pearson’s correlation analysis, hierarchical clustering implemented in the stats package, principal component analysis (PCA) and logistic regression. Hierarchical clustering of the immune cells, diseases and individuals were conducted based on Pearson’s correlations, Euclidian distances and regression coefficients of the two-dimensional matrix of the normalised immune cell counts respectively, of which axes consisted of the samples and immune cell type categorisations. The matrix was regressed by potential confounding factors and immunophenotyping batch effects before hierarchical clustering, including age, sex and the top 10 PCs. The numbers of the defined clusters were sequentially increased until satisfying the following conditions: (1) clusters could distinguish the major immune cell type classification of T cell, B cell and innate immune cells (for immune cell types), (2) each cluster included at least multiple AIRDs (for diseases), (3) multiple clusters included less than 20% of the patients (for patients). Stability of the clustering results were confirmed by repeating de novo clustering by subsampling the randomly selected 95% of the patients from each AIRD (97.4% concordance of the clustering results by×1000 iterations (online supplemental figure 3). The network graphs were visualised by using the igraph package implemented in *R* statistical software (V.1.2.6). Statistical significance of the observed associations was adjusted by using Bonferroni’s correction or Benjamini-Hochberg false-discovery rate (BH-FDR).

### Clinical and genetic associations with immunophenotype in RA

For the patients with RA registered at both the FLOW study and FIRST registry (n=214), clinical phenotypes including biomarkers and Disease Activity Scores (DAS) were obtained at the time of starting and after 24 weeks of the b/tsDMARDs treatment (see details in online supplemental table 3). Genome-wide genotypes of the patients with RA were obtained by using Infinium Asian Screening Array (Illumina). Details of the genotype calling, genotype QC filters, whole-genome genotype imputation and post imputation QC (minor allele frequency >0.5% and Rsq>0.7) are described elsewhere.^30^ We estimated PRS of the subjects based on (1) the transethnic RA case–control GWAS (outside of the HLA region at 6p21)^8^ and (2) the GWAS on RA-interstitial lung disease (ILD),^31^ using PRS-CSx^32^ by adjusting potential population stratification using the PCA components.

## Results

### Cohort-wide Immunophenotyping of the patients with AIRD

Our study enrolled the 1088 patients affected with any of the 11 AIRDs registered at the FLOW study, a prospective cohort with longitudinal follow-up of the clinical information, including RA, SLE, SSc, AAV, IIM, psoriasis, IgG_4_RD, MCTD, AS, SjS and GCA, as well as the 64 controls (table 1).^10 11^ We analysed immunophenotypes by multicolor flow cytometry of PBMCs obtained from the participants according to the Human Immunology Protocol (online supplemental table 1 and online supplemental figure 1).^28^ To normalise potential batch effects, we developed and applied an automated gating and counting pipeline of the immune cells. This advantageously enabled standardisation of immunophenotyping assays across multiple diseases with a cohort-wide scalability. After QC to remove the samples with low quality or technical difficulty in flow cytometry sorting, immunophenotypes were obtained from the 947 subjects as normalised counts of the 46 immune cell types (RA, n=285; SLE, n=170; SSc, n=131; AAV, n=94; IIM, n=77; psoriasis, n=35; IgG4RD, n=35; MCTD, n=22; AS, n=13; SjS, n=20; GCA, n=11; controls, n=54; table 1 and detailed definitions of the immune cell types in online supplemental table 2.

### Immune cell type network identified by the cohort-wide immunophenotyping

**Figure 1:**
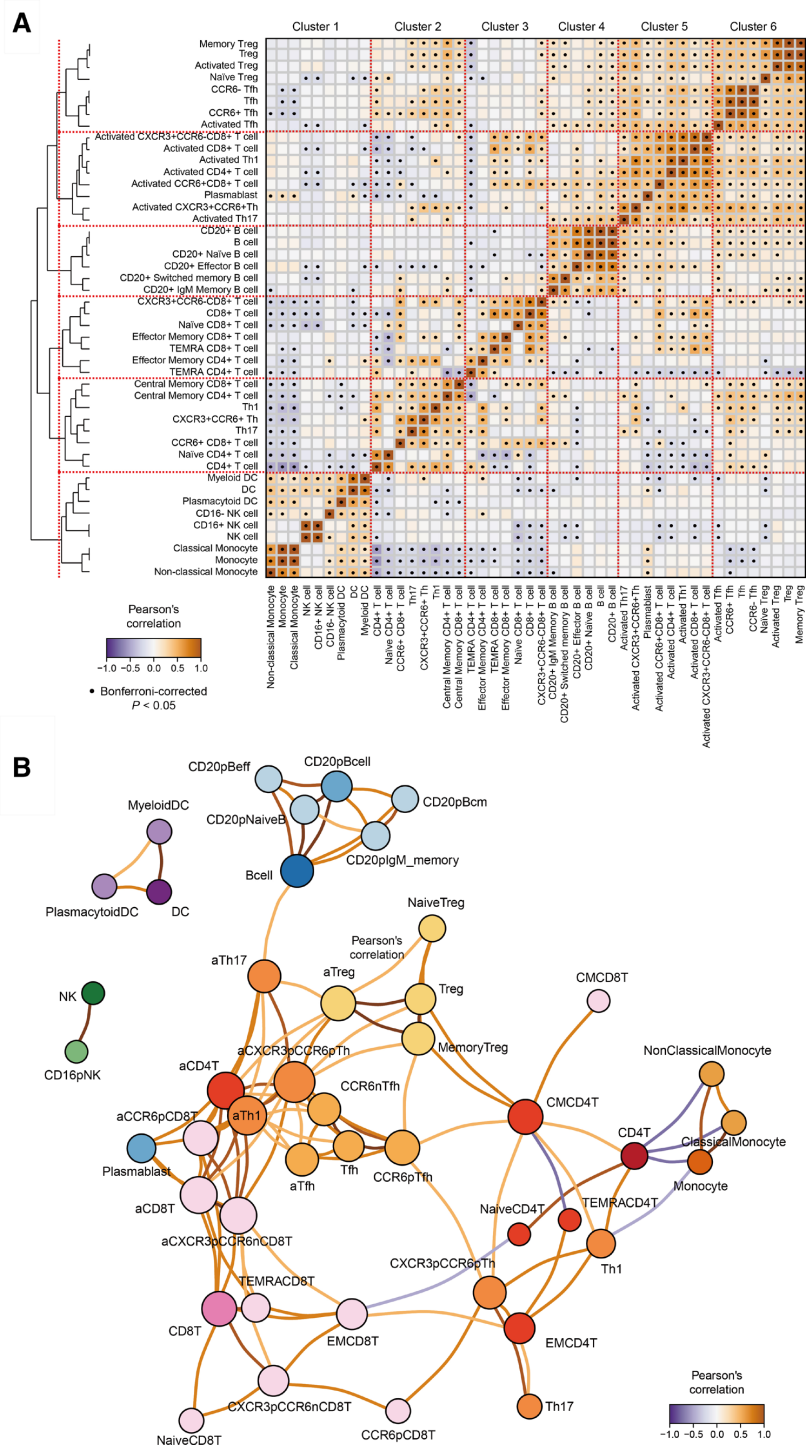
Immunophenotype network across immune cell types. (A) Pairwise correlation of the normalised counts of the 46 immunophenotypes across the patients with AIRD. Cell types were ordered according to the hierarchical clustering analysis. Positive and negative correlations are indicated in red and blue, respectively. Significant correlations satisfied Bonferroni’s multiple testing for the number of the cell type pairs are highlighted (α=0.05). (B) Correlation network of the immunophenotypes. Positive and negative correlations are indicated in red and blue, respectively. Correlations with Pearson’s correlation coefficient |*r*|>0.4 are indicated. AIRD, autoimmune rheumatic disease; DC, dendritic cell.

We first conducted unsupervised hierarchical clustering of the 46 immune cell type abundance defined by the cohort-wide immunophenotyping. The FLOW study recruited the patients with AIRD at initial disease diagnosis or before intensive treatment after disease flare. Therefore, immunophenotypes of the patients with AIRD would reflect primary active states of the disease. We obtained the six major immune cell type clusters. They consisted of the four clusters of T cell subsets (clusters 1, 2, 4, 5), B cell subsets (cluster 3) and innate immune cell subsets (natural killer cells (NKs), DCs and monocytes; cluster 6; figure 1A). The T cell clusters mostly corresponded to those of (1) regulatory T cells (Treg) and follicular helper T (Tfh) cells (cluster 1), (2) activated T cells (cluster 2), (3) CD8+T cells (cluster 4) and (5) CD4+T cells and other T cells (cluster 5). These clusters were concordant to the prior knowledge on peripheral immune cell classifications and their biological kinships,^13^ supporting robust validity in methodological strategy and data quality of the immunophenotyping. Plasmablasts belonged to the activated T cell cluster differentially from other B cell subsets. This may reflect differentiation induction of B cells into plasmablasts accelerated by activated T cells.

Network analysis based on pairwise correlation matrix across immune cell type abundance visualised cell type connections across the patients with AIRD. Tight connection within the major immune cell types of T cells, B cells, DCs, NKs and monocytes were obtained (figure 1B). Again, the network analysis characterised close connection of plasmablasts with activated T cells (eg, activated CD4+, CD8+ and CCR6+CD8+ T cells) rather than with other B cells. We observed negative correlation between CD4+T cells and monocyte lineages, reflecting dominant balance in abundance between these two major cell fractions in peripheral immune cells.

### Cross-autoimmune disease network in sight of immunophenotypes

**Figure 2:**
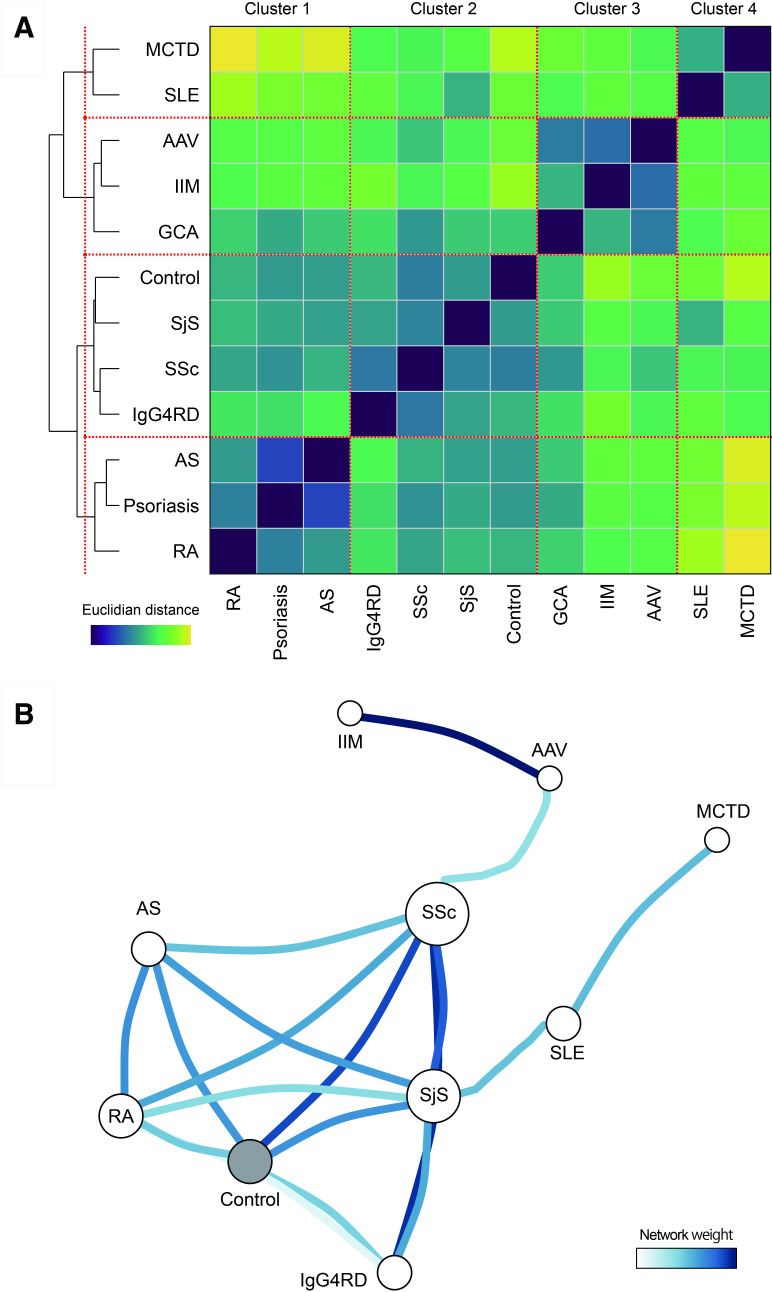
Immunophenotype network across AIRDs. (A) Pairwise Euclidian distance across the AIRDs. AIRDs were ordered according to the hierarchical clustering analysis. Euclidian distances are coloured as in the legend. (B) Euclidian distance network of the AIRDs. Network weights were defined as inverses of the Euclidian distances. Network weights more than one-third of the maximum weight are indicated. AAV, ANCA related vasculitis; AIRD, across autoimmune rheumatic disease; AS, ankylosing spondylitis; GCA, giant cell aortitis; IIM, idiopathic inflammatory myopathy; MCTD, mixed connective tissue disease; RA, rheumatoid arthritis; SjS, Sjogren’s syndrome; SLE, systemic lupus erythematosus; SSc, systemic sclerosis.

We then conducted unsupervised hierarchical clustering of the 11 AIRDs based on pairwise Euclidian distances across AIRDs calculated from principal components (PCs) of the 46 immune cell types. We obtained the four major AIRD clusters, consisting of SLE and MCTD (cluster 1), AAV, IIM, GCA (cluster 2), controls, SjS, SSc, IgG4RD (cluster 3) and AS, psoriasis, RA (cluster 4; figure 2A). Network analysis visualised connections across the AIRDs. In particular, we observed tight positive correlations (1) between SLE and MCTD and (2) between AAV and IIM, which represented relatively similar immunophenotype features within these AIRD pairs (figure 2B).

### Immunophenotype network disentangled immune cell type-specificity of AIRDs

**Figure 3:**
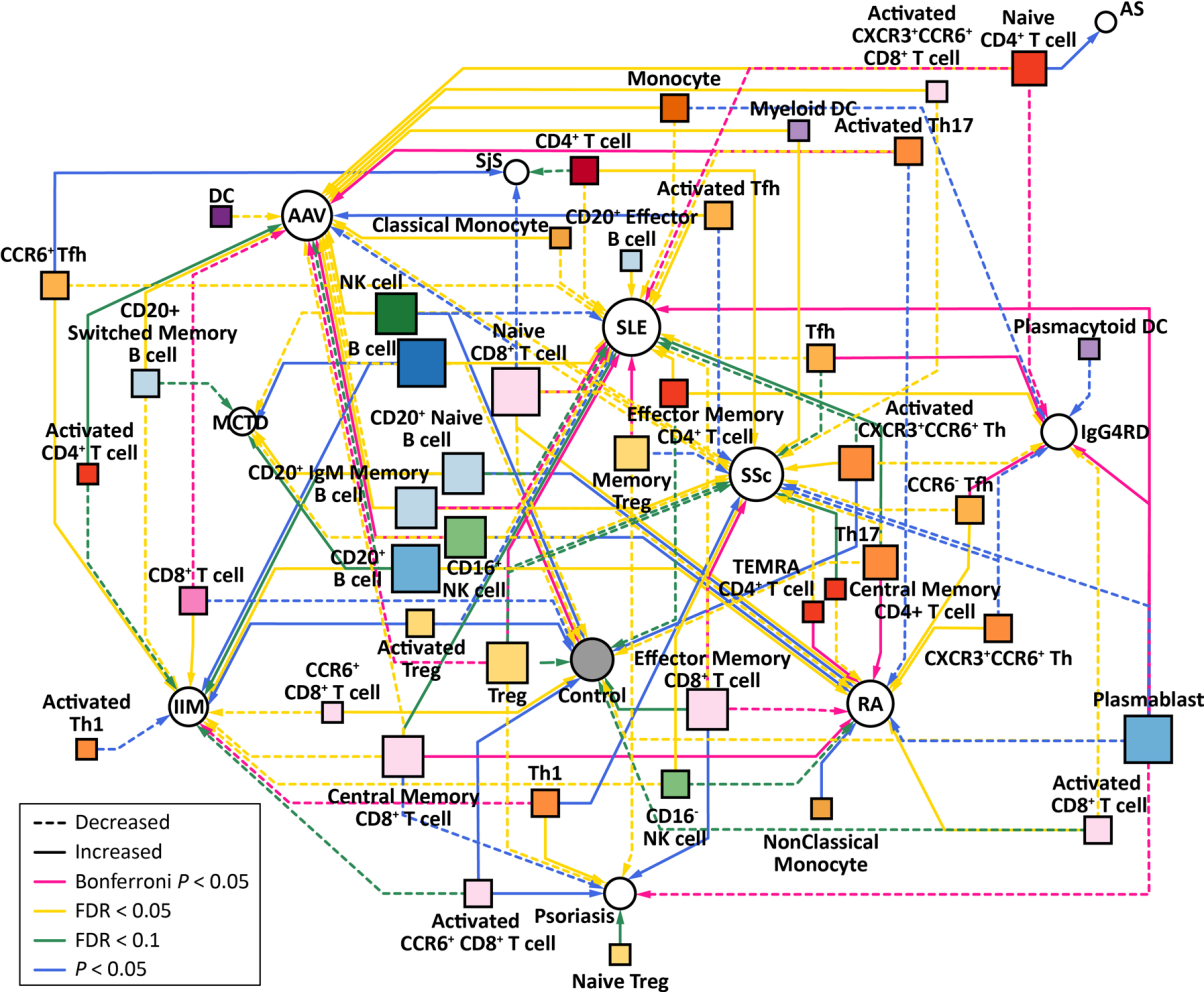
Immunophenotype network across AIRDs and immune cell types. AIRD-immune cell type associations based on backward-forward stepwise logistic regression adjusted by sex were visualised as a network plot. Associations that satisfied Bonferroni’s correction (α=0.05), BH-FDR (<5%, 10%), and nominal significance (p<0.05), were shown as edges with the corresponding colours as indicated in the legend. Dotted and solid lines represent decreasing and increasing directional dosages of the immune cells on AIRDs, respectively. AAV, ANCA related vasculitis; AIRD, across autoimmune rheumatic disease; AS, ankylosing spondylitis; BH-FDR, Benjamini-Hochberg false-discovery rate; SLE, systemic lupus erythematosus; SSc, systemic sclerosis.

To disentangle underlying immunophenotype features defining the AIRD clusters, we constructed immune-phenotype network across the 11 AIRDs and the 46 immune cell types (figure 3). This network visualised immune cell type-specificity of AIRDs, providing immunological explanations of the AIRD clusters obtained in figure 2.

MCTD is characterised as Raynaud’s phenomenon, anti-U1-RNP autoantibody and multiple symptoms of AIRDs,^33^ and the obtained results indicate tight connections in the network with SLE. Enhancement of activated T cell subsets characterised these two diseases. Contrarily, SSc belonged to the same cluster as IgG_4_RD and SjS. IgG_4_RD is a novel disease entity characterised by serum IgG_4_ elevation and tissue infiltration by IgG_4_-positive plasma cells, followed by symptoms related to multiple AIRDs including SjS and SSc.^34 35^ Increase of effector memory CD4+T cells, Tfh cells, and plasmablasts and decrease of naïve CD4+T cells distinguished this cluster. Clinical manifestations of AAV and IIM are diverse,^36^ so their relatively shared immunophenotype was out of expectation. Increase of B cells and monocytes were commonly observed. RA, psoriasis, AS were clustered together, and characterised with autoimmune joint arthritis and shared clinical features such as indication of anti-TNF therapy.^37 38^ These diseases demonstrated as decrease of activated T cells, Treg, Tfh cells, plasmablasts and monocytes. Abnormality of neutrophil extracellular traps has been reported to play a role in the pathogenesis of SLE and AAV.^36 39 40^ We note that this study did not investigate neutrophils, which are not included in PBMCs.

In summary, immunophenotype-based clustering reflected shared clinical features within the defined AIRD clusters, as well as their heterogeneity across the clusters. Underlying immune cell type-specificity prescribes the clusters’ characteristics.

### Patient-level clustering of AIRDs based on immunophenotypes

**Figure 4:**
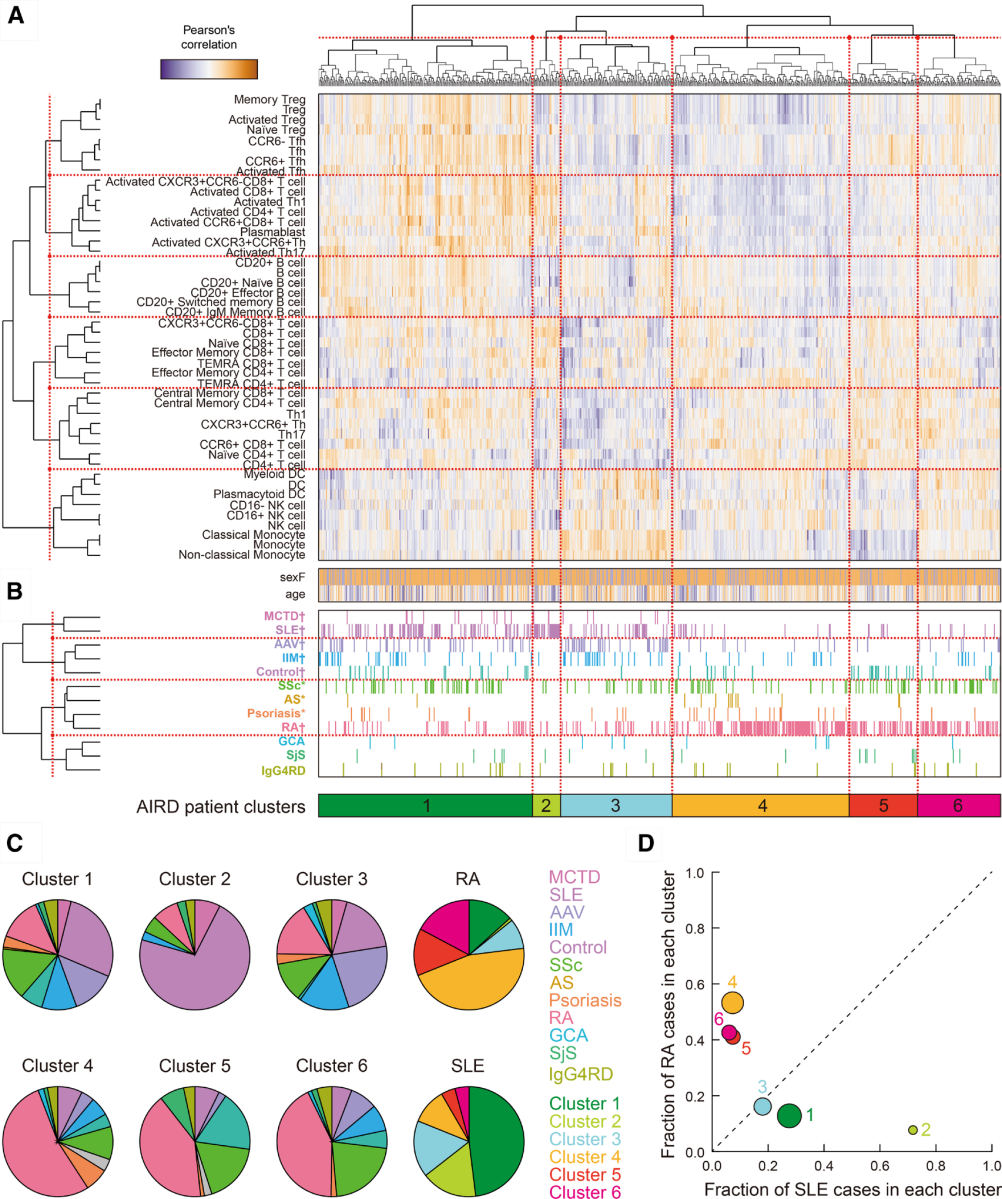
Patient-level AIRD clustering. (A) Unsupervised clustering of individual patients with AIRD according to the immunophenotypes. (B) Clustering of AIRD based on patient-level clustering results. AIRDs with significantly heterogeneous patient distributions across the clusters were highlighted. †Bonferroni’s correction (α=0.05) and *p<0.05. (C) Pie charts showing proportions of the AIRDs within each cluster, and those of the clusters within RA and SLE. (D) A co-plot of the fractions of the patients with RA and SLE within each cluster. AAV, ANCA-related vasculitis; AIRD, across autoimmune rheumatic disease; AS, ankylosing spondylitis; GCA, giant cell aortitis; IIM, idiopathic inflammatory myopathy; MCTD, mixed connective tissue disease; RA, rheumatoid arthritis; SjS, Sjogren’s syndrome; SLE, systemic lupus erythematosus; SSc, systemic sclerosis.

Motivated by successful de novo classifications of AIRDs based on immune cell type specificity, we then conducted unsupervised clustering of individual patients from a cohort-wide immunophenotypes without prior clinical information other than age and sex. Hierarchical clustering based on PCs obtained the major six patient clusters (figure 4A). We assessed patient characteristics separately for each AIRD and found conspicuous heterogeneity in the patient distributions across the clusters for most of the AIRDs (p<0.05/12=0.0042 for MCTD, SLE, AAV, IIM, control, RA; p<0.05 for SSc, AS and psoriasis). Hierarchical clustering of AIRDs based on the patient-level distributions again demonstrated similar AIRD classifications to those obtained in the disease-level clustering in figure 2A (eg, tight connection between AAV and IIM and among AS, psoriasis and RA; figure 4B). Thus, patient-driven clusters of AIRDs could reflect underlying immune cell type specificity of AIRDs, as well as their shared and distinct biological backgrounds, which were identified through our analysis mentioned in the previous sections.

We then assessed fractions of the AIRDs to each patient cluster. We particularly focused on RA and SLE (n=285 and 170, respectively), the two representative AIRDs which consist of the largest sample sizes in our immunophenotype registry with significantly heterogeneous distributions of the patients across the clusters (figure 4C). We found that the clusters 1–3 showed higher fraction of the patients with SLE than in the patients with RA. In contract, the clusters 4–6 showed higher fractions in RA than in SLE (figure 4D). This briefly projects general differentiation of the patients with AIRD to those with RA- or SLE-like immunophenotypes. Namely, our data-driven analysis empirically confirmed the well-recognised clinical observations that RA and SLE are very different diseases.^1 2^ Of note, relatively small proportions of the patients with RA or SLE (23.2% or 18.8%) belonged to the non-RA or non-SLE-like clusters. Discussions on patient heterogeneity and treatment strategies in RA or SLE have been long-standing.^41^ This result provides a piece of empirical evidence of disease subtypes in a single AIRD from the viewpoint of the patient-oriented immunophenotypes.

### Immune cell type and clinical manifestations of the RA patient clusters

To examine how individual patients’ immunophenotypes define clinical characteristics of the heterogeneous AIRD subsets, we conducted association studies between clinical information and patient clusters. We focused on the patients with RA, the AIRD with the largest sample size in our datasets. Further, parallel registration of the patients with RA in another clinical cohort of the FIRST registry enabled us to investigate detailed longitudinal follow-up including disease activity and treatment responses (online supplemental table 3).^26 27^

**Figure 5:**
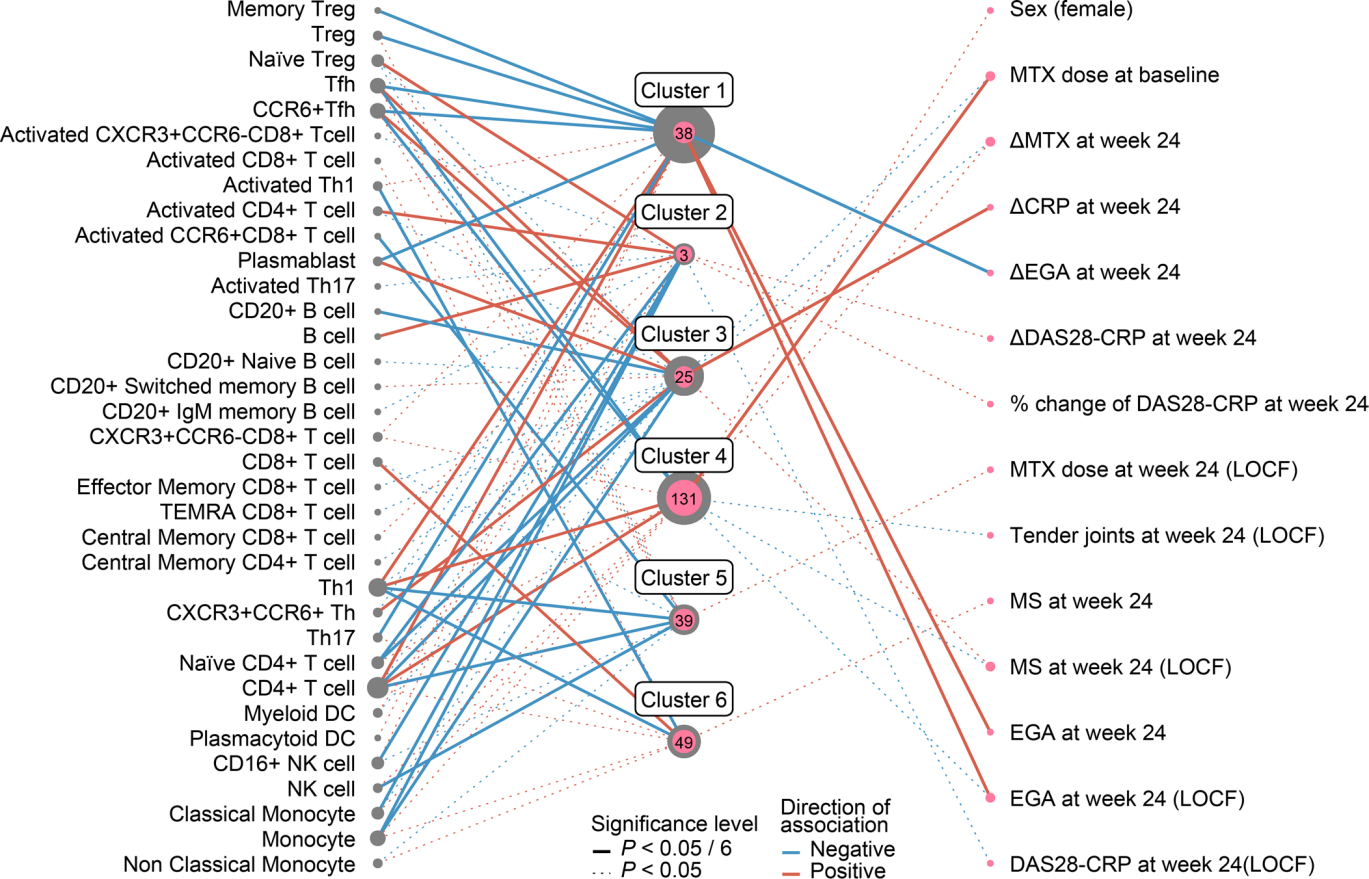
Immune cell type and clinical information associations with the RA patient clusters. Immune cell types and clinical information with significant associations with the RA patient clusters are indicated. Outer and inner circles represent the AIRD and RA patient sample sizes belonging to the clusters, respectively. Edges represent significance levels and directional associations as in the legend. The Δ values show improvement in the disease activity values after treatment (ie, larger positive Δ values corresponding to better treatment responses). The list and values of the clinical information is available in online supplemental tables 3 and 4. AIRD, across autoimmune rheumatic disease; CRP, C reactive protein; DAS-28, Disease Activity Score-28; RA, rheumatoid arthritis.

Each cluster demonstrated specific clinical features (figure 5, online supplemental tables 3 and 4). We did not observe apparent differences of disease durations and female-male ratios across the clusters (FDR>0.05), or treatment choices of the four major biologics categories (TNFα inhibitors, JAK inhibitors, CTLA4-Ig and IL6R inhibitors; p>0.23). As for the patients with RA in the cluster 1, the largest AIRD patient cluster with SLE-like immune-phenotypes, were associated with decreased activated Tregs, Tfh cells, plasmablasts and Th17 cells, and increased Th1 cells and CD4+T cells. The cluster 1 showed average disease activity at enrolment before treatment. However, this cluster was associated with poor treatment response in Evaluator’s global assessment (EGA) after 24 weeks of treatment, showing treatment resistance. Majority of the patients with RA belonged to the cluster 4, the cluster with RA-like immune-phenotypes. The patients with RA in this cluster were characterised as high methotrexate dosage at baseline but showed improvement in EGA and morning stiffness after 24 weeks, presenting its initially severe disease activity but better treatment response. Associated immunophenotypes of the cluster 4 were similar to those of the cluster 1, but negative associations with the T regs were specific to the cluster 1. This may explain treatment resistant feature of the cluster 1, as well as its SLE-like characteristics. We note that no differences in anticitrullinated protein antibody (ACPA) and rheumatoid factor (RF) positivity across the clusters (on average 74.3% and 90.0%, respectively). ACPA positivity is known as a clinical biomarker to predict joint destruction progression and organ failures.^42^ Immunophenotypes could reflect clinical heterogeneity in disease activity as well as treatment response beyond these classical biomarkers. As for the patients with SLE, We observed significant differences of the renal diseases across the SLE patient clusters (FDR<0.005). Lower renal diseases were observed in the clusters 4 and 5 where RA-like immunophenotypes were dominant (16.0%), when compared with the other clusters (46.2%) (online supplemental table 5).

10.1136/ard-2023-224537.supp2 Supplementary data

### Human genetics involvements in clinical and immunological manifestations of RA

**Figure 6:**
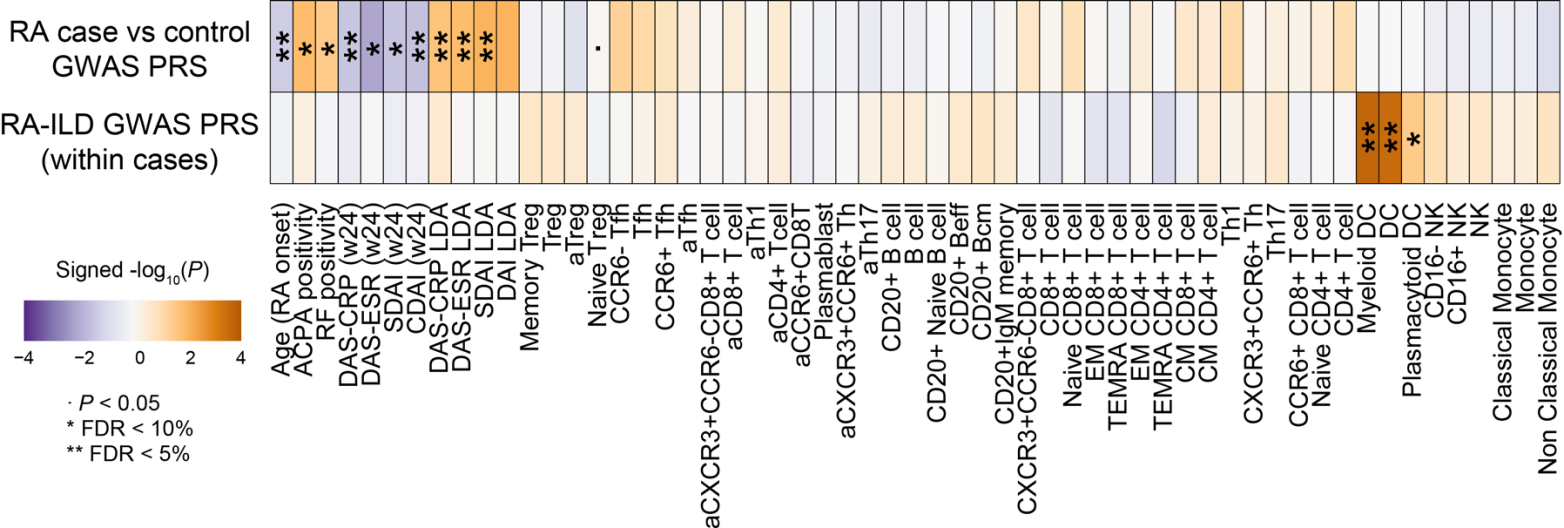
Associations of PRS with clinical and immunological information. Associations of the PRS based on RA case-control GWAS^8^ and within RA case GWAS for RA-ILD^31^ with clinical information and immunophenotypes of the cell types are indicated. Red and blue colours represent positive and negative associations as in the legend, respectively. FDR, false-discovery rate; GWAS, genome-wide association studies; ILD, interstitial lung disease; RA, rheumatoid arthritis; PRS, Polygenic Risk Score.

The aetiopathogenesis of the AIRDs is in part human genetics,^29 43^ which is considered as another promising tool of data-driven approach to decipher disease backgrounds. However, how genetics affect clinical and immunological manifestations within the patients with AIRD after disease onset has been elusive. To investigate human genetics involvements in clinical and immunological manifestations of RA, we conducted an association study of PRS, a genome-wide aggregation of disease risk variants of the individuals to predict phenotype risk,^44 45^ with clinical information and immunophenotypes of the patients with RA registered in the FIRST registry^26 27^ (n=123; figure 6).

We first assessed PRS based on the latest cross-population RA case-control GWAS,^8^ a promising surrogate to predict RA onset susceptibility. RA case–control PRS was associated with younger age of RA onset, autoantibody positivity (ACPA and RF), higher disease activity (DAS-C reactive protein, DAS-ESR, Simple Disease Activity Index and DAI), and poorer treatment responses after 24 weeks (BH-FDR<0.10). Negative associations with decrease of naïve Treg were observed (p<0.05). Thus, PRS based on RA disease onset risk could also be useful to predict disease activity and prognosis.

We then assessed PRS based on the stratified GWAS within the RA cases. We adopted the Japanese GWAS of RA-ILD,^31^ one of the clinically important complications with high morbidity and mortality.^46^ While RA-ILD PRS did not significantly associate with clinical manifestations, we observed positive associations with increased DCs (myeloid DCs, DCs and plasmacytoid DCs; BH-FDR<0.10). While detailed aetiology or RA-ILD has been fully investigated, involvement of DCs in ILD in the rheumatic disease model have been reported.^47^ Our results provided additional supportive evidence in the human RA patients.

## Discussions

In this study, we evaluated the large-scale and cohort-based immunophenotype dataset over 1000 individuals and 11 AIRDs with deep and longitudinal clinical information. De novo clustering of immune cell types and AIRDs successfully constructed classifications consistent with prior knowledge on immunological features of AIRDs. We obtained immune cell type spectra specific to each AIRD or shared across different AIRDs. Further, this provided novel insights into disease aetiology and their underlying networks as well. As an insightful observation, we observed close network between MCTD and SLE. In contrast, SSc was clustered together with SjS and IgG_4_RD, AIRDs characterised with tissue fibrosis and autoantibody production. This may motivate potential utility of the treatment targeting B cell lineages for such AIRDs. Nevertheless, our findings are based on peripheral immune cells, and further investigation using the lesion tissue is warranted. Detailed immunocharacterisation specifically focusing on each AIRD and longitudinal clinical information would also be a next step. While our cohort focuses on primary status of the AIRDs, we admit potential impact of treatment choices and prescription behaviour on immunophenotypes of the patient. Sex-dependent differences in immunological and clinical features are also of interests. While we showed stability, further validation of the hierarchical clustering results would be warranted.

Individual patient-level clustering simplified the patients with AIRD into several clusters, where RA-like and SLE-like ones were exclusively dominant. The immunological abnormalities found in the patients with RA and SLE reflect their clinical differences (eg, incidence of SLE-like symptoms in patients with RA as consequence of anti-TNF therapy). Since we focused on primary disease status of AIRD and did not handle secondary SjS as an independent disease, elucidation of immune profile shift driven by secondary SjS would be also warranted. In general, patients are likely to manifest more than one autoimmune condition. Such complex conditions may make patient diagnosis based on immunophenotyping challenging.^48^

In-depth analysis of the patients with RA revealed that such patient clusters differentially defined clinical heterogeneity in disease activity and longitudinal treatment responses. Of interest, the patients with RA with SLE-like immunophenotypes such as decreased Treg were characterised as treatment resistance. We note that the FLOW study mostly focused on recruitment of the early-onset patients with RA with high disease activity before treatment (bio-naïve for >75% of the patients). This mitigated biases from patient heterogeneity in disease status or treatment strategies and empowered the analysis differently from previous studies. Our results had advantageous messages that cohort-wide and cross-disease immune-phenotyping would be required to pinpoint such heterogeneous patient subtypes existing within the single disease. Given that our approach identified the disease subtypes along with immune cell type specificity, this facilitated personalised treatment strategy or novel drug discovery targeting such key immune cell types.^49^

Inborn human genetics highlighted with PRS showed associations with dynamics in clinical manifestations and immunophenotypes. Previous studies reported values of PRS to predict disease onset in early stages of life,^44 45^ but how PRS could benefit patient stratification and personalised treatment strategy after onset is not yet widely validated. We showed that PRS from both case–control GWAS and within-case stratified GWAS could contributed to fulfil such missing pieces. Further, associations between GWAS-derived PRS and immunophenotypes suggested candidate key immune cell types implicated in disease aetiology (eg, DCs for RA-ILD in figure 6), proposing a novel biological utility of PRS.

While our study achieved translayer integrations across immunophenotypes, clinical information and genetics, further involvement of multilayer omics resources should be warranted to more finely characterise AIRDs. This may include metabolome,^50^ proteome,^51^ microbiome,^52^ virome^53^ and somatic alterations.^44^ One potential limitation of immunophenotyping is being unable to know transcriptomic and epigenetic dynamics within the immune cells. Recent development of single cell technologies could complement this layer, and its application in the field of AIRDs are expected.^54^ Nevertheless, expansion of the target diseases into wider ranges of AIRDs and immune-related human complex phenotypes is also the future step. Dynamic and longitudinal effects of treatment on immunophenotypes is of clinical interests. We note that the number of the control subjects (n=64) were relatively smaller than those of the AIRDs. Enlargement of the control subjects is desirable to further validate our findings on AIRDs. Finally, while our study provided robust associations of immunophenotype with AIRD clinical features, we need more evidence to reliably predict patients’ future clinical outcomes.

In summary, our study provided a piece of evidence that large-scale immunophenotypes could characterise AIRDs, define underlying within-disease subsets, and profile individual patients, which would be useful for implementing personalised medicine of AIRDs.

## Data Availability

Data are available on reasonable request. The data are available on the request to the corresponding authors.

## References

[1] Smolen JS , Aletaha D , Barton A , et al . Rheumatoid arthritis. Nat Rev Dis Primers 2018;4:18001. 10.1038/nrdp.2018.1 29417936

[2] Kaul A , Gordon C , Crow MK , et al . Systemic lupus erythematosus. Nat Rev Dis Primers 2016;2:16039. 10.1038/nrdp.2016.39 27306639

[3] Aletaha D , Kerschbaumer A , Kastrati K , et al . Consensus statement on blocking Interleukin-6 receptor and Interleukin-6 in inflammatory conditions: an update. Ann Rheum Dis 2023;82:773–87. 10.1136/ard-2022-222784 35953263

[4] Lin CMA , Cooles FAH , Isaacs JD . Precision medicine: the precision gap in rheumatic disease. Nat Rev Rheumatol 2022;18:725–33. 10.1038/s41584-022-00845-w 36216923

[5] Guthridge JM , Wagner CA , James JA . The promise of precision medicine in rheumatology. Nat Med 2022;28:1363–71. 10.1038/s41591-022-01880-6 35788174 PMC9513842

[6] Tanaka Y , Luo Y , O’Shea JJ , et al . Janus kinase-targeting therapies in rheumatology: a mechanisms-based approach. Nat Rev Rheumatol 2022;18:133–45. 10.1038/s41584-021-00726-8 34987201 PMC8730299

[7] Okada Y , Wu D , Trynka G , et al . Genetics of rheumatoid arthritis contributes to biology and drug discovery. Nature 2014;506:376–81. 10.1038/nature12873 24390342 PMC3944098

[8] Ishigaki K , Sakaue S , Terao C , et al . Multi-ancestry genome-wide Association analyses identify novel genetic mechanisms in rheumatoid arthritis. Nat Genet 2022;54:1640–51. 10.1038/s41588-022-01213-w 36333501 PMC10165422

[9] Shirai Y , Nakanishi Y , Suzuki A , et al . Multi-trait and cross-population genome-wide association studies across autoimmune and allergic diseases identify shared and distinct genetic component. Ann Rheum Dis 2022;81:1301–12. 10.1136/annrheumdis-2022-222460 35753705 PMC9380494

[10] Ma X , Nakayamada S , Kubo S , et al . Expansion of T follicular helper-T helper 1 like cells through epigenetic regulation by signal transducer and activator of transcription factors. Ann Rheum Dis 2018;77:1354–61. 10.1136/annrheumdis-2017-212652 29853448

[11] Ueno M , Miyagawa I , Miyazaki Y , et al . Efficacy and safety of guselkumab and adalimumab for pustulotic arthro-osteitis and their impact on peripheral blood immunophenotypes. Arthritis Res Ther 2022;24:240. 10.1186/s13075-022-02934-3 36303202 PMC9609190

[12] Kubo S , Nakayamada S , Yoshikawa M , et al . Peripheral Immunophenotyping identifies three subgroups based on T cell heterogeneity in lupus patients. Arthritis Rheumatol 2017;69:2029–37. 10.1002/art.40180 28605137

[13] Nakayamada S , Kubo S , Yoshikawa M , et al . Differential effects of biological Dmards on peripheral immune cell phenotypes in patients with rheumatoid arthritis. Rheumatology (Oxford) 2018;57:164–74. 10.1093/rheumatology/kex012 28371836

[14] Kay J , Upchurch KS . ACR/EULAR 2010 rheumatoid arthritis classification criteria. Rheumatology 2012;51(suppl 6):vi5–9. 10.1093/rheumatology/kes279 23221588

[15] Petri M , Orbai A-M , Alarcón GS , et al . Derivation and validation of the systemic lupus international collaborating clinics classification criteria for systemic lupus erythematosus. Arthritis & Rheumatism 2012;64:2677–86. 10.1002/art.34473 22553077 PMC3409311

[16] Asano Y , Jinnin M , Kawaguchi Y , et al . Diagnostic criteria, severity classification and guidelines of systemic sclerosis. J Dermatol 2018;45:633–91. 10.1111/1346-8138.14162 29687465

[17] Tanimoto K , Nakano K , Kano S , et al . Classification criteria for polymyositis and dermatomyositis. J Rheumatol 1995;22:668–74. 7791161

[18] Fujibayashi T , Sugai S , Miyasaka N , et al . Revised Japanese criteria for Sjögren’s syndrome (1999): availability and validity. Mod Rheumatol 2004;14:425–34. 10.3109/s10165-004-0338-x 24387718

[19] Watts R , Lane S , Hanslik T , et al . Development and validation of a consensus methodology for the classification of the ANCA-associated vasculitides and polyarteritis nodosa for epidemiological studies. Ann Rheum Dis 2007;66:222–7. 10.1136/ard.2006.054593 16901958 PMC1798520

[20] Masi AT , Hunder GG , Lie JT , et al . The American college of rheumatology 1990 criteria for the classification of Churg-Strauss syndrome (allergic granulomatosis and angiitis). Arthritis Rheum 1990;33:1094–100. 10.1002/art.1780330806 2202307

[21] Leavitt RY , Fauci AS , Bloch DA , et al . The American college of rheumatology 1990 criteria for the classification of Wegener’s granulomatosis. Arthritis Rheum 1990;33:1101–7. 10.1002/art.1780330807 2202308

[22] Umehara H , Okazaki K , Masaki Y , et al . Comprehensive diagnostic criteria for Igg4-related disease (Igg4-RD). Mod Rheumatol 2012;22:21–30. 10.1007/s10165-011-0571-z 22218969

[23] Taylor W , Gladman D , Helliwell P , et al . Classification criteria for psoriatic arthritis: development of new criteria from a large international study. Arthritis Rheum 2006;54:2665–73. 10.1002/art.21972 16871531

[24] Bennett PH , Wood PHN . Population studies of the rheumatic diseases; proceedings of the third international symposium. Excerpta Medica Foundation Amsterdam; 1966

[25] Kasukawa R , Too T , Miyawaki S , et al . Preliminary diagnostic criteria for classification of mixed connective tissue disease. In: Kasukawa R , Sharp GC , eds. Mixed connective tissue diseases and anti-nuclear antibodies 41. Amsterdam: Elsevier, : e7.

[26] Kawabe A , Nakano K , Kubo S , et al . Differential long-term retention of biological disease-modifying antirheumatic drugs in patients with rheumatoid arthritis by age group from the FIRST registry. Arthritis Res Ther 2020;22:136. 10.1186/s13075-020-02233-9 32513309 PMC7282084

[27] Ochi S , Sonomoto K , Nakayamada S , et al . Preferable outcome of Janus kinase inhibitors for a group of difficult-to-treat rheumatoid arthritis patients: from the FIRST registry. Arthritis Res Ther 2022;24:61. 10.1186/s13075-022-02744-7 35232462 PMC8886884

[28] Maecker HT , McCoy JP , Nussenblatt R . Standardizing immunophenotyping for the human immunology project. Nat Rev Immunol 2012;12:191–200. 10.1038/nri3158 22343568 PMC3409649

[29] Sakaue S , Kanai M , Tanigawa Y , et al . A cross-population atlas of genetic associations for 220 human phenotypes. Nat Genet 2021;53:1415–24. 10.1038/s41588-021-00931-x 34594039 PMC12208603

[30] Sonehara K , Kimura Y , Nakano Y , et al . A common deletion at Bak1 reduces enhancer activity and confers risk of intracranial germ cell tumors. Nat Commun 2022;13:4478. 10.1038/s41467-022-32005-9 35918310 PMC9346128

[31] Shirai Y , Honda S , Ikari K , et al . Association of the Rpa3-Umad1 locus with interstitial lung diseases complicated with rheumatoid arthritis in Japanese. Ann Rheum Dis 2020;79:1305–9. 10.1136/annrheumdis-2020-217256 32737115 PMC7509520

[32] Ruan Y , Lin Y-F , Feng Y-C , et al . Author correction: improving polygenic prediction in ancestrally diverse populations. Nat Genet 2022;54. 10.1038/s41588-022-01144-6 35789324

[33] Tanaka Y , Kuwana M , Fujii T , et al . Diagnostic criteria for mixed connective tissue disease (MCTD): from the Japan research committee of the ministry of health, labor, and welfare for systemic autoimmune diseases. Modern Rheumatology 2021;31:29–33. 10.1080/14397595.2019.1709944 31903831

[34] Kubo S , Nakayamada S , Tanaka Y . Immunophenotype involved in Igg4-related disease. Mod Rheumatol 2019;29:226–30. 10.1080/14397595.2018.1537962 30334637

[35] Matsui S . Igg4-related respiratory disease. Mod Rheumatol 2019;29:251–6. 10.1080/14397595.2018.1548089 30474465

[36] Nakazawa D , Masuda S , Tomaru U , et al . Author correction: pathogenesis and therapeutic interventions for ANCA-associated vasculitis. Nat Rev Rheumatol 2019;15:123. 10.1038/s41584-019-0168-z 30655606

[37] Mauro D , Thomas R , Guggino G , et al . Ankylosing spondylitis: an autoimmune or autoinflammatory disease Nat Rev Rheumatol 2021;17:387–404. 10.1038/s41584-021-00625-y 34113018

[38] FitzGerald O , Ogdie A , Chandran V , et al . Psoriatic arthritis. Nat Rev Dis Primers 2021;7:59. 10.1038/s41572-021-00293-y 34385474

[39] Kaplan MJ . Role of neutrophils in systemic autoimmune diseases. Arthritis Res Ther 2013;15:219. 10.1186/ar4325 24286137 PMC3978765

[40] Villanueva E , Yalavarthi S , Berthier CC , et al . Netting neutrophils induce endothelial damage, infiltrate tissues, and expose Immunostimulatory molecules in systemic lupus erythematosus. J Immunol 2011;187:538–52. 10.4049/jimmunol.1100450 21613614 PMC3119769

[41] Kubo S , Nakayamada S , Tanaka Y . Baricitinib for the treatment of rheumatoid arthritis and systemic lupus erythematosus: a 2019 update. Expert Rev Clin Immunol 2019;15:693–700. 10.1080/1744666X.2019.1608821 30987474

[42] van Delft MAM , Huizinga TWJ . An overview of autoantibodies in rheumatoid arthritis. J Autoimmun 2020;110:102392. 10.1016/j.jaut.2019.102392 31911013

[43] Okada Y , Eyre S , Suzuki A , et al . Genetics of rheumatoid arthritis: 2018 status. Ann Rheum Dis 2019;78:446–53. 10.1136/annrheumdis-2018-213678 30530827

[44] Namba S , Saito Y , Kogure Y , et al . Common germline risk variants impact somatic alterations and clinical features across cancers. Cancer Research 2023;83:20–7. 10.1158/0008-5472.CAN-22-1492 36286845 PMC9811159

[45] Khera AV , Chaffin M , Aragam KG , et al . Genome-wide polygenic scores for common diseases identify individuals with risk equivalent to monogenic mutations. Nat Genet 2018;50:1219–24. 10.1038/s41588-018-0183-z 30104762 PMC6128408

[46] Hyldgaard C , Hilberg O , Pedersen AB , et al . A population-based cohort study of rheumatoid arthritis-associated interstitial lung disease: comorbidity and mortality. Ann Rheum Dis 2017;76:1700–6. 10.1136/annrheumdis-2017-211138 28611082

[47] Sendo S , Saegusa J , Okano T , et al . Cd11B+Gr-1 dim tolerogenic dendritic cell-like cells are expanded in interstitial lung disease in SKG mice. Arthritis Rheumatol 2017;69:2314–27. 10.1002/art.40231 28805019

[48] Hahn BH et al . Systemic lupus erythematosus. In: Jameson JR , ed. Harrison’s Principles of Internal Medicine, 21th edition. Columbus: McGraw-Hill, 2022.

[49] Namba S , Konuma T , Wu K-H , et al . A practical guideline of genomics-driven drug discovery in the era of global biobank meta-analysis. Cell Genom 2022;2:100190. 10.1016/j.xgen.2022.100190 36778001 PMC9903693

[50] Kishikawa T , Maeda Y , Nii T , et al . Increased levels of plasma nucleotides in patients with rheumatoid arthritis. Int Immunol 2021;33:119–24. 10.1093/intimm/dxaa059 32866240 PMC7846180

[51] Saevarsdottir S , Stefansdottir L , Sulem P , et al . Multiomics analysis of rheumatoid arthritis yields sequence variants that have large effects on risk of the seropositive subset. Ann Rheum Dis 2022;81:1085–95. 10.1136/annrheumdis-2021-221754 35470158 PMC9279832

[52] Kishikawa T , Maeda Y , Nii T , et al . Metagenome-wide association study of gut microbiome revealed novel aetiology of rheumatoid arthritis in the Japanese population. Ann Rheum Dis 2020;79:103–11. 10.1136/annrheumdis-2019-215743 31699813 PMC6937407

[53] Tomofuji Y , Kishikawa T , Maeda Y , et al . Prokaryotic and viral genomes recovered from 787 Japanese gut metagenomes revealed microbial features linked to diets, populations, and diseases. Cell Genom 2022;2:100219. 10.1016/j.xgen.2022.100219 36778050 PMC9903723

[54] Edahiro R , Shirai Y , Takeshima Y , et al . Single-cell analyses and host genetics highlight the role of innate immune cells in COVID-19 severity. Nat Genet 2023;55:753–67. 10.1038/s41588-023-01375-1 37095364 PMC10181941

