# Supplementary material for: Extracting immunological and clinical heterogeneity across autoimmune rheumatic diseases by cohort-wide immunophenotyping
Source: Ann Rheum Dis. 2023 Oct 30;83(2):242–52. doi: 10.1136/ard-2023-224537 (PMC10850648; doi:10.1136/ard-2023-224537)
Supplement: Supplementary data [file ard-2023-224537supp001.pdf]

Supplementary Information

Extracting immunological and clinical heterogeneity across autoimmune rheumatic diseases by cohort-wide immuno-phenotyping

Tanaka H and Okada Y et al.

Corresponding to: Prof. Yukinori Okada, MD, PhD (yuki-okada@m.u-tokyo.ac.jp) and Prof. Yoshiya Tanaka, MD, PhD (tanaka@med.uoeh-u.ac.jp).

Supplementary Method of the immuno-phenotyping.

Informed consent having been obtained, peripheral blood samples were collected and promptly subjected to multicolor flow cytometry analysis. Peripheral blood mononuclear cells (PBMCs) were isolated from these samples using lymphocyte isolation medium (ICN/Cappel Pharmaceuticals, Aurora, OH, USA). Subsequently, the PBMCs were resuspended in phosphate-buffered saline (PBS) containing 3% human IgG (Baxter International Inc.) to inhibit Fc receptor activity and prevent nonspecific antibody binding. They were then incubated for 15 minutes at 48°C in a light-free environment, followed by a PBS wash containing 1% BSA.

The assessment of background fluorescence was accomplished through suitable isotype- and fluorochrome-matched control monoclonal antibodies. After antibody staining, the PBMCs were subjected to multicolor flow cytometry analysis using the FACSVerse (BD Biosciences, San Jose, CA, USA). The antibodies used for antibody staining are detailed in **Supplementary Table 1**.

For immune cell subset phenotyping, the comprehensive eight-color flow cytometry analysis protocol proposed by the National Institutes of Health/Federation of Clinical Immunology Societies (HIP protocol) was followed, with minor modification made to enable the detection of Tfh cells. **Supplementary Figure 1** provides a detailed representation of the gating strategy, which outputs the 46 immune cell types of the participants.

The phenotypes of immune cell subsets were defined based on the Human Immunology Protocol of comprehensive 8-color flow cytometric analysis proposed by National Institutes of Health/Federation of Clinical Immunology Sciences, with minor modifications for detecting Tfh cells. Details of the gating strategy are in **Supplementary Figure 2**.

To normalize potential batch effects among the samples and assays, we developed a pipeline to apply automated counting of the immune cells using R Bioconductor packages of openCyto (version 2.2.0). The list of the 46 cell types is shown in **Supplementary Table 2**. Counts of each cell and sample were log transformed after normalized by the total viable PBMC counts (i.e., sum of the CD4+ T cells, CD3-CD19+ B cells, CD3-CD14-CD19-CD20-CD56+ NK cells, CD3-CD14-CD19-CD20-CD56-HLA-DR+ dendric cells, and CD14+ monocytes). Details of our immuno-phentyping protocol are also described in the following references.

1. Nakayamada, S. et al. Differential effects of biological DMARDs on peripheral immune cell phenotypes in patients with rheumatoid arthritis. ***Rheumatology*** (Oxford) 57, 164–174 (2018).
2. Kubo, S. et al. Peripheral Immunophenotyping Identifies Three Subgroups Based on T Cell Heterogeneity in Lupus Patients. ***Arthritis Rheumatol*** 69, 2029–2037 (2017).

Tanaka H, *et al. Ann Rheum Dis* 2024; 83:242–252. doi: 10.1136/ard-2023-224537

Supplementary Table 1. Details of the panels used in immuno-phenotyping.

Panel	Antibody	Production company	Catalog number
T lymphocytes subset	FITC Mouse anti-human CD28	BD	555728
T lymphocytes subset	PE Mouse anti-human CD197 (CCR7)	BD	560765
T lymphocytes subset	PerCP/Cy5.5 anti-human CD4	BioLegend	300530
T lymphocytes subset	PE-Cy™7 Mouse anti-human CD45RA	BD	560675
T lymphocytes subset	APC Mouse anti-human CD38	BD	555462
T lymphocytes subset	APC-H7 Mouse anti-human CD8	BD	561423
T lymphocytes subset	V450 Mouse anti-human CD3	BD	561812
T lymphocytes subset	V500 Mouse anti-human HLA-DR	BD	561224
Regulately T lymphocytes subset	Fixable Viability Dye eFluor™ 520	Thermo Fisher	65-0867-18
Regulately T lymphocytes subset	PE Mouse anti-human CD25	BD	555432
Regulately T lymphocytes subset	PerCP/Cy5.5 anti-human CD4	BioLegend	300530
Regulately T lymphocytes subset	PE-Cy™7 Mouse anti-human CD194	BD	557864
Regulately T lymphocytes subset	CD127 Monoclonal antibody, APC	eBioscience	17-1278-42
Regulately T lymphocytes subset	APC-H7 Mouse anti-human CD45RO	BD	561137
Regulately T lymphocytes subset	V450 Mouse anti-human CD3	BD	561812
Regulately T lymphocytes subset	V500 Mouse anti-Human HLA-DR	BD	561224
Helper T lymphocytes subset	Fixable Viability Dye eFluor™ 520	Thermo Fisher	65-0867-18
Helper T lymphocytes subset	PE Mouse anti-human CD183	BD	550633
Helper T lymphocytes subset	PerCP/Cy5.5 anti-human CD4	BioLegend	300530
Helper T lymphocytes subset	PE-Cy™7 Mouse anti-human CD196 (CCR6)	BD	560620
Helper T lymphocytes subset	APC Mouse anti-human CD38	BD	555462
Helper T lymphocytes subset	APC-H7 Mouse anti-human CD8	BD	561423
Helper T lymphocytes subset	V450 Mouse anti-human CD3	BD	561812
Helper T lymphocytes subset	V500 Mouse anti-human HLA-DR	BD	561224
B lymphocytes subset	Fixable Viability Dye eFluor™ 520	Thermo Fisher	65-0867-18
B lymphocytes subset	PE Mouse anti-human CD24	BD	555428
B lymphocytes subset	PerCP-Cy5.5 Mouse anti-human CD19	BD	340951
B lymphocytes subset	PE/Cy7 anti-human CD27	BioLegend	302838
B lymphocytes subset	APC Mouse anti-human CD38	BD	555462
B lymphocytes subset	APC-H7 Mouse anti-human CD20	BD	560853
B lymphocytes subset	V450 Mouse anti-human CD3	BD	561812
B lymphocytes subset	V500 Mouse anti-human IgD	BD	561490
Monocytes, dendritic cells, natural killer cells	Fixable Viability Dye eFluor™ 520	Thermo Fisher	65-0867-18
Monocytes, dendritic cells, natural killer cells	PE Mouse anti-human CD56	BD	555516
Monocytes, dendritic cells, natural killer cells	PerCP-Cy™5.5 Mouse anti-human CD123	BD	558714
Monocytes, dendritic cells, natural killer cells	PE-Cy™7 Mouse anti-human CD11c	BD	561356
Monocytes, dendritic cells, natural killer cells	APC Mouse anti-human CD16	BD	561304
Monocytes, dendritic cells, natural killer cells	APC-H7 Mouse anti-human CD3	BD	560176
Monocytes, dendritic cells, natural killer cells	APC-H7 Mouse anti-human CD19	BD	560177
Monocytes, dendritic cells, natural killer cells	APC-H7 Mouse anti-human CD20	BD	560853
Monocytes, dendritic cells, natural killer cells	V450 Mouse anti-human CD14	BD	560349
Monocytes, dendritic cells, natural killer cells	V500 Mouse anti-human HLA-DR	BD	561224
Follicular helper T lymphocytes subset	FITC Mouse anti-human CD28	BD	555728
Follicular helper T lymphocytes subset	Human CXCR5 PE-conjugated Antibody	R&D	FAB190P-10[illegible]
Follicular helper T lymphocytes subset	PerCP-Cy™5.5 Mouse anti-human CD183	BD	560832
Follicular helper T lymphocytes subset	PE-Cy™7 Mouse anti-human CD196 (CCR6)	BD	560620
Follicular helper T lymphocytes subset	CD278 (ICOS) Monoclonal Antibody, APC	ThermoFisher	17-9948-42
Follicular helper T lymphocytes subset	APC-H7 Mouse anti-human CD69	BD	560737
Follicular helper T lymphocytes subset	V450 Mouse anti-human CD3	BD	561812
Follicular helper T lymphocytes subset	V500 Mouse anti-human CD4	BD	560768

Tanaka H, *et al. Ann Rheum Dis* 2024; 83:242–252. doi: 10.1136/ard-2023-224537

Supplementary Table 2. Abbreviations for the immune cell types.

Abbreviation	Immune cell type
CD4T	$CD4^{+}$ T cell
NaiveCD4T	$CD4^{+}$ Tcell
TEMRACD4T	$CD4^{+}$ Tcell
EMCD4T	$CD4^{+}$ Tcell
CMCD4T	$CD4^{+}$ Tcell
aCD4T	$CD4^{+}$ Tcell
Th1	T helper 1 cell
aTh1	Activated T helper 1 cell
Th17	T helper 17 cell
aTh17	Activated T helper 17 cell
CXCR3pCCR6pTh	$CXCR3^{+}CCR6^{+}$ T helper cell
aCXCR3pCCR6pTh	Activated $CXCR3^{+}CCR6^{+}$ T helper cell
Tfh	T follicular helper cell
aTfh	Activated T follicular helper cell
CCR6nTfh	$CCR6^{-}$ T follicular helper cell
CCR6pTfh	$CCR6^{+}$ T follicular helper cell
Treg	T regulatory cell
aTreg	Activated T regulatory cell
NaiveTreg	Naïve T regulatory cell
MemoryTreg	Memory T regulatory cell
CD8T	$CD8^{+}$ T cell
NaiveCD8T	Naïve $CD8^{+}$ T cell
TEMRACD8T	Terminally Differentiated Effector Memory $CD8^{+}$ T cell
EMCD8T	Effector Memory $CD8^{+}$ T cell
CMCD8T	Central Memory $CD8^{+}$ T cell
aCD8T	Activated $CD8^{+}$ T cell
CXCR3pCCR6nCD8T	$CXCR3^{+}CCR6^{-}$ $CD8^{+}$ T cell
CCR6pCD8T	$CCR6^{+}$ $CD8^{+}$ T cell
aCXCR3pCCR6nCD8T	Activated $CXCR3^{+}CCR6^{-}$ $CD8^{+}$ T cell
aCCR6pCD8T	Activated $CCR6^{+}$ $CD8^{+}$ T cell
Bcell	B cell
CD20pBcell	$CD20^{+}$ B cell
CD20pIgM_memory	$CD20^{+}$ IgM Memory B cell
CD20pNaiveB	$CD20^{+}$ Naïve B cell
CD20pBeff	$CD20^{+}$ Effector B cell
CD20pBcm	$CD20^{+}$ Switched Memory B cell
Plasmablast	Plasmablast
NK	Natural Killer cell
CD16pNK	$CD16^{+}$ Natural Killer cell
CD16nNK	$CD16^{-}$ Natural Killer cell
DC	Dendritic cell
PlasmacytoidDC	Plasmacytoid Dendritic cell
MyeloidDC	Myeloid Dendritic cell
Monocyte	Monocyte
ClassicalMonocyte	Classical Monocyte
NonClassicalMonocyte	Non-Classical Monocyte

Tanaka H, *et al. Ann Rheum Dis* 2024; 83:242–252. doi: 10.1136/ard-2023-224537

Supplementary Table 3. Clinical information of the RA patient clusters.

Clinical information of the RA patients	Total	Cluster 1	Cluster 2	Cluster 3	Cluster 4	Cluster 5	Cluster 6
No. samples	214	26	3	17	104	28	36
No. samples (genotyped)	73	10	0	5	37	11	10
Age	62.0 (13.2)	61.6 (13.9)	58.3 (15.6)	65.4 (14.8)	60.8 (13.6)	61.2 (9.5)	65.3 (13.3)
Female [%]	84.1	76.9	100.0	70.6	89.4	82.1	80.6
Disease duration, median [IQR]	3.75 [1.41 - 9.8]	7.50 [1.25-11.75]	0 [0-10.0]	2.0 [1.0-12.0]	4.0 [1.0-9.25]	2.0 [1.0-5.25]	2.5 [1.0-13.75]
ACPA positive [%]	76.7	84.0	66.7	70.6	76.7	81.5	71.4
RF positive [%]	77.9	76.0	100.0	76.5	78.8	75.0	77.8
PSL usage [%]	21.5	11.5	33.3	35.3	23.1	17.9	19.4
MTX dose [mg/week]	10.0 (5.8)	8.6 (6.6)	3.3 (5.8)	6.5 (6.3)	11.1 (5.7)	11.9 (4.6)	8.4 (6.3)
PSL dose [mg/day]	1.3 (3.2)	0.4 (1.1)	20.0 (34.6)	2.6 (5.3)	1.2 (3.5)	0.5 (1.3)	0.7 (2.0)
MS [min]	119.9 (103.3)	128.3 (102.4)	93.3 (127.4)	136.3 (97.8)	108.4 (99.9)	129.1 (113.9)	134.2 (105.9)
GH [mm]	50.6 (23.8)	48.4 (25.0)	46.3 (12.6)	53.2 (27.3)	50.4 (21.1)	52.4 (30.7)	50.7 (24.7)
Pain-VAS [mm]	52.1 (24.5)	53.0 (27.7)	42.5 (21.9)	53.7 (30.1)	50.0 (22.7)	55.2 (30.9)	54.9 (20.1)
EGA [mm]	40.9 (19.5)	38.4 (20.8)	42.0 (20.9)	40.8 (21.9)	39.5 (19.0)	43.5 (19.9)	44.4 (18.7)
TJ	8.5 (6.8)	8.0 (6.4)	13.0 (3.6)	11.1 (7.1)	8.2 (6.9)	7.6 (7.1)	8.7 (6.4)
SJ	7.2 (5.2)	6.7 (6.3)	13.7 (11.2)	8.6 (5.6)	7.2 (5.6)	5.3 (3.0)	8.1 (4.0)
CRP [mg/dL]	2.2 (3.0)	1.7 (1.8)	1.7 (1.7)	3.8 (4.5)	2.1 (3.3)	2.1 (2.3)	2.1 (3.2)
ESR [mm/hr]	51.3 (32.5)	47.9 (33.5)	69.0 (43.9)	59.2 (34.3)	48.2 (32.8)	51.4 (32.9)	57.5 (29.0)
CDAI	24.8 (12.6)	23.3 (14.5)	35.5 (15.2)	27.1 (13.1)	24.6 (12.7)	22.5 (11.7)	26.4 (11.3)
SDAI	27.0 (13.7)	25.0 (15.1)	37.2 (15.5)	30.3 (14.7)	26.8 (14.2)	24.5 (11.9)	28.5 (12.0)
DAS28-CRP	4.7 (1.3)	4.5 (1.4)	5.5 (0.9)	5.2 (1.5)	4.6 (1.3)	4.5 (1.3)	4.8 (1.1)
DAS28-ESR	5.4 (1.3)	5.2 (1.5)	6.4 (0.3)	5.9 (1.5)	5.3 (1.3)	5.2 (1.4)	5.7 (1.1)
ΔGH at week 24	22.9 (30.4)	13.9 (28.4)	27.0 (18.5)	23.5 (41.5)	21.4 (29.4)	31.4 (27.1)	26.2 (32.9)
ΔPGA at week 24	26.8 (27.7)	19.6 (29.2)	29.0 (9.9)	29.8 (37.2)	26.0 (25.6)	30.5 (28.6)	30.1 (29.1)
ΔEGA at week 24	32.1 (21.2)	16.6 (25.1)	34.7 (28.0)	37.5 (18.4)	33.3 (20.8)	35.0 (18.3)	34.6 (22.3)
ΔTJ at week 24	6.9 (6.5)	4.5 (8.8)	13.0 (3.6)	9.6 (6.5)	7.2 (6.4)	5.4 (5.4)	7.3 (6.0)
ΔSJ at week 24	6.6 (5.3)	5.7 (6.3)	13.7 (11.2)	8.5 (5.5)	6.5 (5.8)	4.8 (2.9)	7.6 (4.5)
ΔCRP at week 24	1.9 (3.1)	1.3 (1.6)	1.7 (1.7)	4.9 (5.1)	1.7 (3.2)	1.7 (2.4)	1.9 (3.5)
ΔESR at week 24	27.3 (30.6)	19.0 (31.0)	38.0 (26.0)	40.0 (31.0)	26.0 (29.0)	24.0 (33.0)	33.0 (33.0)
ΔCDAI at week 24	18.8 (12.4)	13.1 (18.2)	32.8 (18.5)	20.8 (6.9)	19.2 (12.6)	16.8 (9.6)	21.0 (11.7)
% change of CDAI at week 24	71.2 (30.4)	36.9 (94.7)	87.8 (17.7)	79.4 (15.3)	75.4 (19.7)	77.5 (20.1)	73.5 (31.1)
ΔSDAI at week 24	20.6 (13.5)	14.3 (18.4)	34.5 (18.9)	24.7 (9.2)	21.1 (14.1)	18.4 (10.1)	22.3 (12.2)
% change of SDAI at week 24	71.9 (30.8)	41.2 (90.1)	88.0 (17.5)	80.7 (13.9)	75.2 (22.2)	77.5 (19.7)	74.4 (30.3)
ΔDAS28-CRP at week 24	2.5 (1.4)	1.9 (1.8)	4.1 (1.2)	3.3 (1.6)	2.5 (1.4)	2.2 (1.3)	2.6 (1.4)
% change of DAS28-CRP at week 24	50.3 (24.3)	36.9 (41.3)	74.4 (11.5)	59.5 (17.8)	51.2 (22.2)	47.7 (23.5)	52.9 (23.0)
ΔDAS28-ESR at week 24	2.6 (1.5)	1.9 (2.0)	4.3 (1.7)	3.0 (1.1)	2.6 (1.5)	2.2 (1.3)	2.8 (1.5)
% change of DAS28-ESR at week 24	44.3 (23.7)	32.5 (38.9)	66.4 (25.8)	51.3 (15.3)	46.0 (20.3)	39.4 (30.2)	46.3 (21.2)
CDAI remission at week 24	29.0	30.8	66.7	17.6	27.9	35.7	27.8
CDAI LDA at week 24	65.0	57.7	100.0	47.1	73.1	64.3	52.8
SDAI remission at week 24	29.9	30.8	66.7	17.6	29.8	35.7	27.8
SDAI LDA at week 24	65.9	57.7	100.0	47.1	74.0	64.3	55.6
DAS28-CRP remission at week 24	50.5	46.2	100.0	35.3	55.8	50.0	41.7
DAS28-CRP LDA at week 24	58.9	57.7	100.0	35.3	66.3	50.0	52.8
DAS28-ESR remission at week 24	32.7	26.9	66.7	17.6	35.6	42.9	25.0
DAS28-ESR LDA at week 24	50.0	50.0	66.7	41.2	54.8	50.0	38.9

The values in the table represent mean, and those in parenthesis represent SD. RA; rheumatoid arthritis, ACPA; anti-citrinated peptide antibody, RF; rheumatoid factor, PSL; pledonisolone, MTX; methotrexate, MS; morning stiffness, GH; patient's self-assessed general health, VAS; visual analogue scale, EGA; evaluator global disease activity estimate, TJ; tender joint, SJ; swollen joint, CRP; C-reactive protein, ESR; erythrocyte sedimentation rate,DAS28; disease activity score, CDAI; clinical disease activity index, SDAI; simplified disease activity index, LDA; low disease activity.

Supplementary Table 4. Associations of the immune cell types with the patient clusters.

(Supplementary Table 4 is provided as a separate Microsoft Excel sheet)

Tanaka H, *et al. Ann Rheum Dis* 2024; 83:242–252. doi: 10.1136/ard-2023-224537

Supplementary Table 5. ISN/RPS classification of the SLE patient clusters.

ISN/RPS classification of the SLE patients (%)	Total	Cluster 1	Cluster 2	Cluster 3	Cluster 4	Cluster 5	Cluster 6
Without lupus nephritis or any renal involvement	99 (58.2)	45 (54.9)	14 (50.0)	16 (57.1)	14 (77.8)	7 (100.0)	3 (42.9)
Non-investigated renal involvement (e.g., contraindications to renal biopsy)	6 (3.5)	3 (3.7)	3 (10.7)	0 (0.0)	0 (0.0)	0 (0.0)	0 (0.0)
II	10 (5.9)	8 (9.8)	1 (3.6)	1 (3.6)	0 (0.0)	0 (0.0)	0 (0.0)
IIIA/IVA	41 (24.1)	23 (28.0)	10 (35.7)	6 (21.4)	0 (0.0)	0 (0.0)	2 (28.6)
IIIC/IVC (without "active" lesion)	3 (1.8)	0 (0.0)	0 (0.0)	1 (3.6)	2 (11.1)	0 (0.0)	0 (0.0)
V	10 (5.9)	2 (2.4)	0 (0.0)	4 (14.3)	2 (11.1)	0 (0.0)	2 (28.6)
VI	1 (0.6)	1 (1.2)	0 (0.0)	0 (0.0)	0 (0.0)	0 (0.0)	0 (0.0)
Total	170	82	28	28	18	7	7

Tanaka H, *et al. Ann Rheum Dis* 2024; 83:242–252. doi: 10.1136/ard-2023-224537

Supplementary Figure 1. Design and strategy of the study.

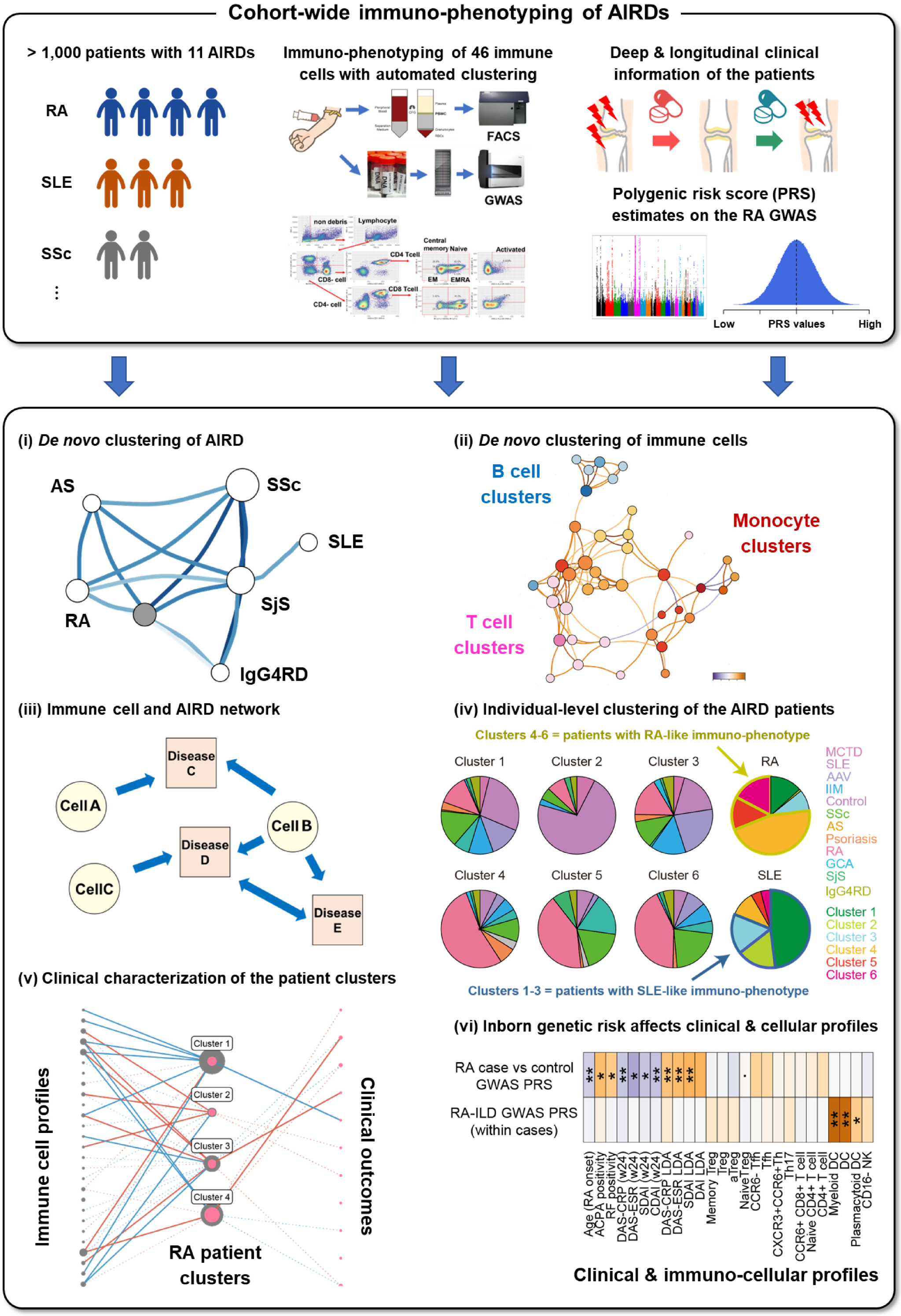

Tanaka H, *et al. Ann Rheum Dis* 2024; 83:242–252. doi: 10.1136/ard-2023-224537

Supplementary Figure 2. Clustering protocols of the immune cell types.

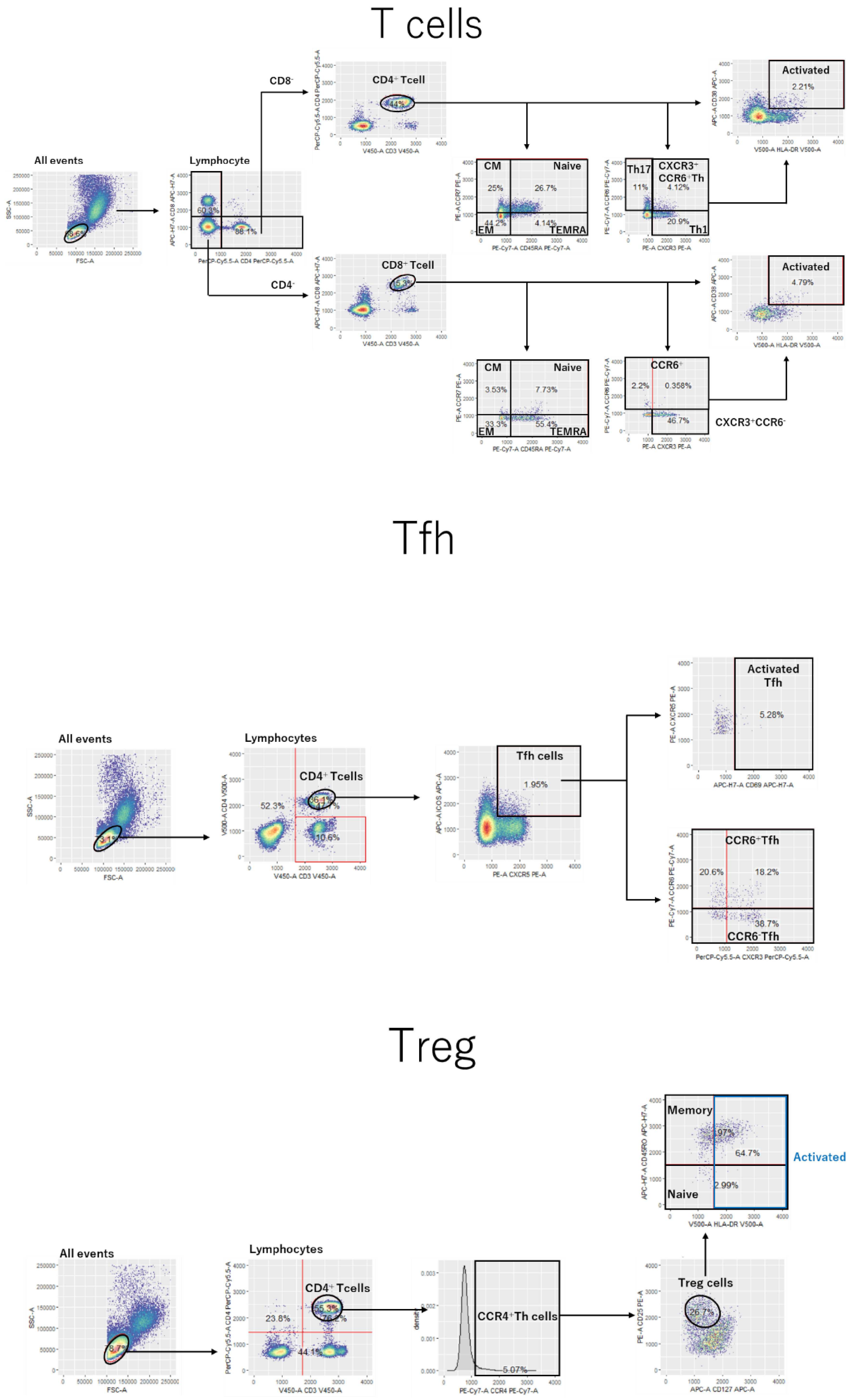

Tanaka H, *et al. Ann Rheum Dis* 2024; 83:242–252. doi: 10.1136/ard-2023-224537

B cells

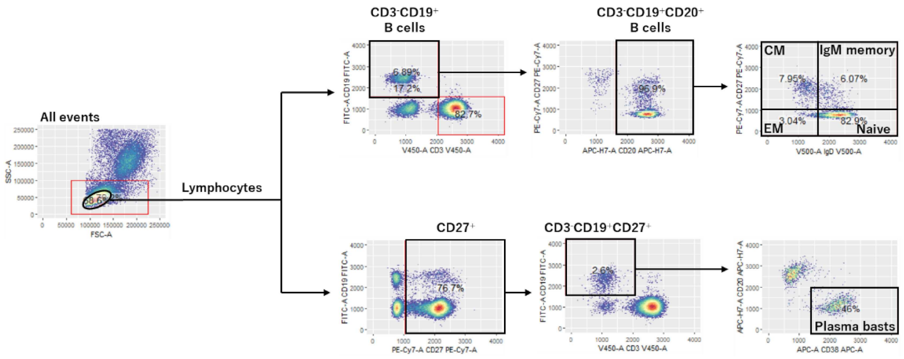

Monocytes/DCs/NK cells

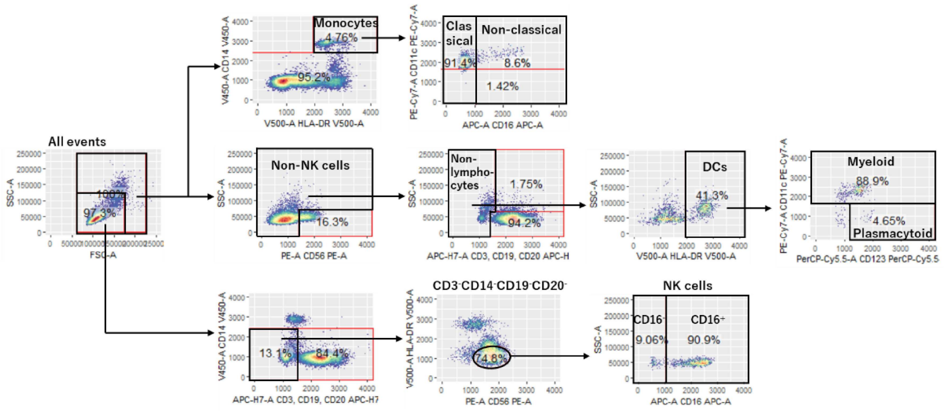

Supplementary Figure 3. Stability of the hierarchical clustering by iterative subsampling.

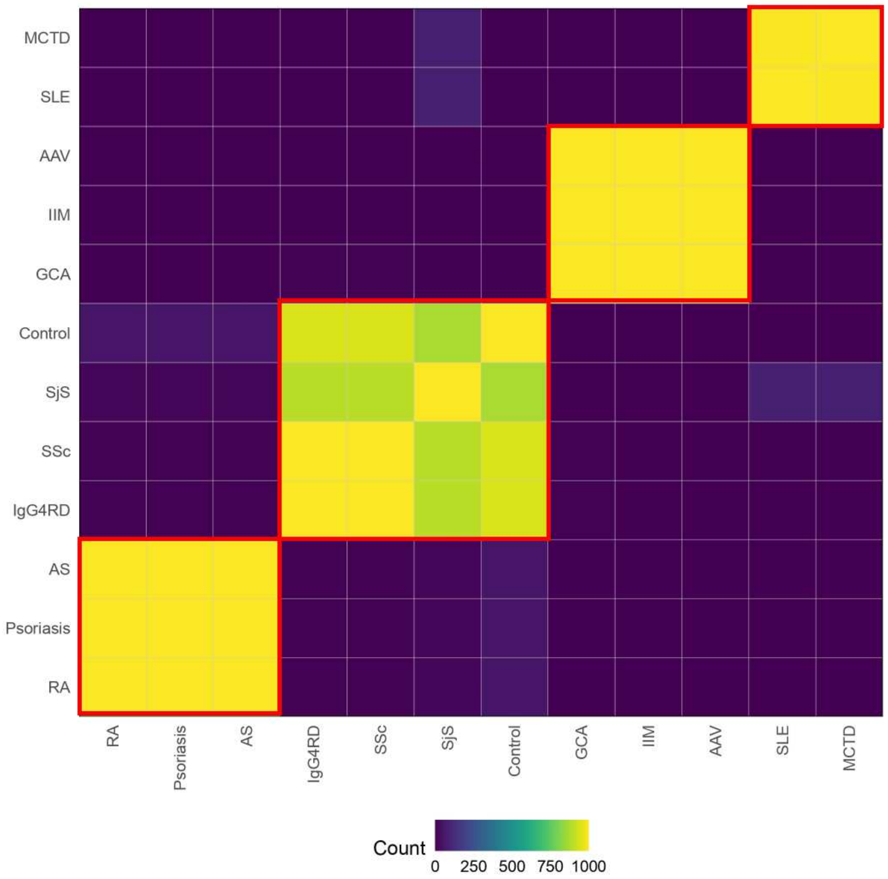

Hierarchical clustering of the randomly subsampled 95% of the patients from each AIRD were repeated by ×1,000 iterations. Concordance in belongings to the initially defined cluster was evaluated by pairwise comparisons of the AIRDs, where the initial clusters were highlighted by red squares.

Tanaka H, *et al. Ann Rheum Dis* 2024; 83:242–252. doi: 10.1136/ard-2023-224537
